# Scoping review of the characteristics assessed by vulnerability indices applied to people experiencing homelessness

**DOI:** 10.1371/journal.pone.0254100

**Published:** 2021-07-09

**Authors:** Jane Currie, Elizabeth Grech, Erin Longbottom, Jasmine Yee, Ruth Hastings, Amy Aitkenhead, Amy Cason, Karin Obrecht

**Affiliations:** 1 Queensland University of Technology Faculty of Health, School of Nursing, Brisbane, Qld, Australia; 2 Homeless Health Service, St Vincent’s Hospital Sydney, New South Wales, Australia; The University of Sydney, AUSTRALIA

## Abstract

**Background:**

The objectives of this scoping review are to investigate the characteristics assessed by existing vulnerability indices and the health outcomes achieved by applying them to people experiencing homelessness. This review forms part of the development and implementation of a novel tool to prioritise people experiencing homelessness for healthcare based on their need and capacity to access healthcare.

**Methods:**

Included papers were primary research, published in the English language, participants were experiencing homelessness and aged over 18 years at the time of the study, a vulnerability index was used in the study, sample size was greater than 30, and the study had a health focus. Databases searched were MEDLINE, Embase, CINAHL, Scopus, PubMed and Web of Science, between January-April 2020. The Joanna Briggs Appraisal criteria were used to quality appraise the included studies. Results were synthesised narratively.

**Results:**

Six papers were included, a total of 27,509 participants. The reported use of the indices varied; they included screening and profiling homeless populations, comparing homeless populations, and two studies evaluated the predictive capacity, reliability and validity of the indices. One vulnerability index focused on screening for human immunodeficiency virus, one used a 50-item index and four used a nine-item index. No direct health outcomes were reported from applying the vulnerability indices. The studies identified limitations of using vulnerability indices, including the potential bias of relying on self-reported data and two studies highlighted the need for further psychometric testing to ensure validity and reliability of the indices.

**Discussion:**

The sample of included studies was small. Vulnerability indices are reportedly a useful and easily accessible method of gaining valuable data on the health status and health needs of people experiencing homelessness. The variety of characteristics included in the vulnerability indices suggests the importance of tailoring vulnerability indices to the needs of the population to which it is to be applied. Conducting appropriate psychometric testing is critical so that an index can be used to accurately inform decision making and accurately prioritise people experiencing homelessness who are most at risk of mortality and morbidity. A specific tool that prioritises people experiencing homelessness for access to health care is not yet available. The review was funded by a St Vincent’s Network Inclusive Health grant.

## Introduction

This scoping review investigates the characteristics assessed by existing vulnerability indices and the health outcomes achieved by applying them to people experiencing homelessness. Vulnerability indices are used to assess mortality and morbidity of people experiencing homelessness in order to prioritise them for housing. Defined further below, vulnerability indices ask a series of questions relating to a person’s health and wellbeing and populate a risk score based on self-reported data, to prioritise people for housing. This review underpins the development of a novel tool to prioritise people experiencing homelessness for healthcare (rather than housing) based on an assessment of their healthcare need and their capacity to access healthcare.

People experiencing homelessness have poorer health outcomes than the general population [1, 2]. Experiencing homelessness places people at greater risk of physical health issues, including musculoskeletal disorders, respiratory tract infections, skin infections, accidental injury and violence, and poor oral health and mental illness are common amongst this cohort [1, 2]. A vulnerability index is a measure of the exposure of a population to some hazard, in this case the development of poorer health outcomes. Research suggests a correlation between housing status and health risk factors, including poor physical health, substance use and mental illness [3–5]. One of the first studies to investigate the relationship between mortality and homelessness was conducted by a homeless service in Boston assessing 558 adults who died between 1993–1998 [6]. Specific main indicators for death were acquired immunodeficiency syndrome (AIDS), symptomatic human immunodeficiency virus (HIV) infection, renal disease and a history of cold-related injury [6]. In other studies from Boston, the all-cause mortality of people under the age of 65 years who were experiencing homelessness was reported as 5–10 times greater [7] and 15 times higher [8] compared to the general population. The increased mortality has been explained by the high exposure to substance use, smoking tobacco and mental illness [9].

There is disproportionate use of acute health services by those experiencing homelessness [9]. People experiencing homelessness more frequently re-present to the emergency department (ED) and have longer lengths of stay once admitted [10, 11] compared to the general population. This review examines the characteristics included in existing vulnerability indices, how they have been applied and any direct health outcomes achieved by applying them. The findings of this review are used to inform, for the first time, the development of a novel tool for use in primary healthcare settings and emergency department settings to prioritise people experiencing homelessness for healthcare.

### Vulnerability index

In the context of homelessness, the first vulnerability index was developed in 2007 by Common Ground, a homelessness service in New York City, for the purpose of assessing medical vulnerability and to prioritise housing for people experiencing homelessness [12]. Based on the findings of earlier research [10], this original vulnerability index provides a framework to prioritise housing need by identifying the most medically vulnerable people experiencing homelessness through a standardised assessment, which quantifies the individual’s risk for mortality. It comprises nine criteria, including length of homelessness (days, months, years), number of hospital admissions and emergency department admissions in a year, age, cirrhosis of the liver, end stage renal disease, history of frost-bite/hypothermia, HIV+/AIDS, mental health, substance use and chronic health conditions. Scores range from 0 to 8, and to receive a score higher than zero, an individual must have experienced at least six months of homelessness [13]. It takes approximately 15 minutes to complete and relies on self-reported data from clients [14]. It has been widely implemented, particularly in the United States (US), where its use has been documented in 62 US communities [15] and in Canada [16].

### Defining Australian homelessness

According to the Australian Bureau of Statistics’ (ABS) definition, homelessness is when a person has no suitable accommodation and lives in an inadequate dwelling, has no or limited/non-extendable tenure, and has no control of/no access to or space for social relations [17]. Homelessness is an umbrella term used to describe four broad population groups 1) Rough/Street sleeping; 2) Supported accommodations (e.g. refuges & crisis accommodation; 3) Short–term accommodation without tenure (e.g. boarding houses, hostel, caravan, couch surfing); and 4) Accommodation in institutional settings (e.g. hospitals, drug and alcohol rehabilitation centres, jail) [18]. Aboriginal and Torres Strait Islander people are over-represented in homelessness in Australia, making up 3% of the total population but 20% of all persons who were experiencing homelessness on Census night in 2016 [17]. The causes of homelessness are extremely complex and include structural factors such as criminal justice system association and poverty along with individual factors such as trauma, substance use, and mental illness [9]. The likelihood of accessing and receiving health care decreases with the complexity and length of homelessness [19].

In Australia, more than 116,000 people are experiencing homelessness [17]. The City of Sydney Bi-Annual Street Count reports 334 people sleeping on the streets and 505 people sleeping in crisis and temporary accommodation [20]. The lifetime prevalence of health conditions among the inner-Sydney homeless population were recorded in the Registry Week survey, 30 November– 2 December 2015 [20]. The most prevalent health conditions reported were hepatitis C, followed by asthma, heart problems and liver disease [20]. Two Australian studies examined emergency service use over two separate time periods (24 months over 2003–4, and one month in 2009), and both reported that people experiencing homelessness had lower rates of access to general practitioners (GPs) than general population [11, 21].

### Rationale for the review

The authors of this review practice at St Vincent’s Hospital Homeless Health Service, Sydney, Australia. Established in 2010, the Homeless Health Service consists of a multidisciplinary team of nurses, doctors, allied health professionals, drug and alcohol clinicians, oral health educators, peer support workers, and Aboriginal health workers. The service provides holistic, multidisciplinary healthcare and support to people experiencing homelessness in the City of Sydney Local Government Area (LGA). To date, the Homeless Health Service has not employed any form of assessment tool to identify clients experiencing homelessness at particular risk of mortality and morbidity. This review is the first stage of a project to develop and implement a tool to prioritise people experiencing homelessness for access to healthcare services based on their health need and their capacity to access healthcare.

## Methods

A scoping review was undertaken to synthesise current evidence on the characteristics included in existing vulnerability indices and their application to people experiencing homelessness [22, 23]. The specific research questions were:

1. In the context of homelessness, what characteristics, health conditions and comorbidities are assessed by existing vulnerability indices?
2. What health outcomes have been achieved by applying vulnerability indices to people experiencing homelessness?

The population of interest was people experiencing homelessness in any country of the world. The intervention was any type of vulnerability indices. The outcomes of interest were the characteristics included in each vulnerability index and any health outcomes achieved from applying a vulnerability index.

### Protocol and registration

A scoping review of the literature was conducted between January and April 2020, reported here in accordance with the Preferred Reporting Items for Systematic Review and Meta-analyses Scoping Review extension (PRISMA-ScR) guidelines [22]. A study protocol was developed and registered initially as a systematic review on PROSPERO (doi: 10.15124/CRD42020166983) in January 2020, and later amended to a scoping review on account of the breadth of the research questions.

**Fig 1:**
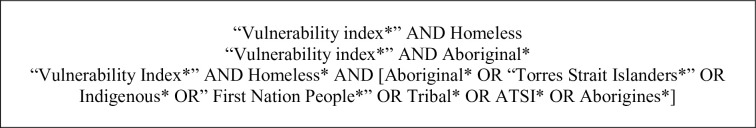
Search terms.

The search strategy was identified by the authorship team, a multidisciplinary team of clinicians and academics with considerable knowledge of health service delivery to people experiencing homelessness. Prior to agreeing to the selected search terms (Fig 1) a scoping review of terms was conducted, which included the terms measure* OR risk factor* and resulted in a diverse range of papers that were non-applicable to the research questions and were therefore excluded. The final search terms are shown in Fig 1 and were used to search in each database, MEDLINE, Embase, CINAHL, Scopus, PubMed, Web of Science. A copy of the search strategy is provided as a S1 File.

A search of prominent authors in the field of homelessness was also undertaken (Conroy, E., O’Connell, J., Parsell, C., Teasdale, M., Baldry, E.). In addition, the search terms *Vulnerability Index AND Homelessness* were searched through Google Scholar. Saturation was reached after the first four pages of results.

### Search & eligibility criteria

All search results (n = 430) were exported to EndNote X8 for eligibility assessment. After removing duplicates (n = 213) the abstracts were screened against the inclusion and exclusion criteria, shown in Table 1, independently by two authors. There were no disagreements between the authors. The remaining 34 studies were read independently in full by at least two authors and screened against the eligibility criteria and then discussed with all authors for consensus.

**Table 1:** Inclusion and exclusion criteria.

Inclusion	Exclusion
Primary research	<30 participants in study (equal to 10% of homeless clients in Sydney catchment area)
Study written in English language	Study participants <18 years old
Study participants experiencing homelessness	Not related to homelessness
Study has a health focus	No vulnerability index used
Peer-reviewed publication	

### Critical appraisal

Given that the results of this scoping review were to be used to underpin the development of a clinical tool, the research team undertook a critical appraisal of the included studies. The critical appraisal was undertaken using the Joanna Briggs Analytical Cross Section Design Appraisal criteria [24]. Two authors (JC, EG) independently appraised each study and compared their findings, any disagreements were resolved through discussion.

### Data charting and synthesis

A data charting form was developed by two of the authors (EG & JC) and reviewed by all authors, there was unanimous agreement in the characteristics extracted from the included studies. The data charting was undertaken independently by two authors (EG & JC) and then findings were compared and discussed. There were no disagreements between the two authors and the findings extracted were extremely similar. Data extraction included author, year, country, participants, study design, results and conclusion. Also extracted from the studies were the characteristics included in each vulnerability index, including the demographic data, health history and health service usage.

## Results

### Selection of sources of evidence

**Fig 2:**
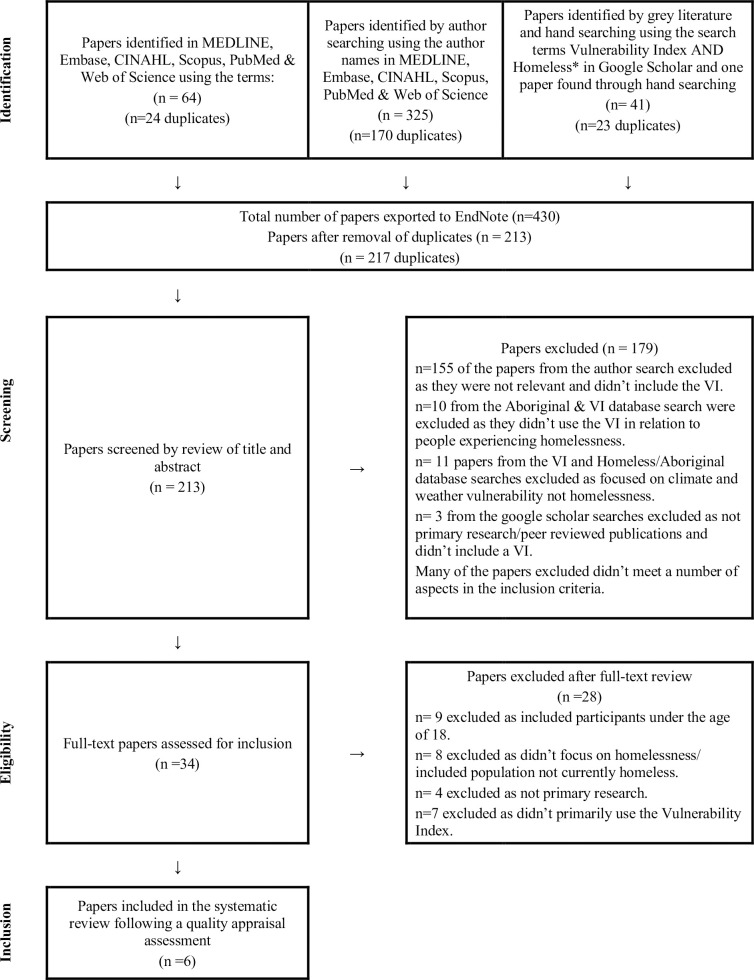
PRISMA diagram.

This scoping review investigated the characteristics assessed by vulnerability indices and the health outcomes achieved by applying vulnerability indices to people experiencing homelessness. There was unanimous agreement that six studies met the inclusion and exclusion criteria and were included in this systematic review. The results of the search process are outlined in Fig 2.

### Critical appraisal of sources of evidence

Overall, the selected papers were of a high quality and therefore none of the studies were removed on the basis of rigour (Table 2). Each study gained ethics/board approval, recruitment processes were well described, and appropriate statistical analysis was conducted. Across all of the studies, three papers clearly met all of the appraisal criteria. In two of the studies the management of confounding variables was unclear [16, 25].

**Table 2:** Critical appraisal.

	Bowie & Lawson (2018) Seattle, US	Brown, Cummings, Lyons, Carrion, & Watson (2018). Midwest, US	Cronley, Petrovich, Spence-Almaguer, & Preble. (2013) Fort Worth, Texas, US	Berbesi, Segura, Cardona, Caicedo, (2017) Medellin, Colombia	Montgomery, Syzmkowiak, Marcus, Howard, Culhane. (2016) 62 communities in the US	Nicholson, Graham, Emery, Schiff, Giacomin, Tanasescu. (2008) Calgary, Canada
Were the criteria for inclusion in the sample clearly defined?	Yes	Yes	Yes	Yes	Yes	Yes
Were the study subjects and the setting described in detail?	Yes	Yes	Yes	Yes	Unclear	Yes
Was the exposure measured in a valid and reliable way?	Yes	Yes	Yes	Yes	Yes	Yes
Were objective, standard criteria used for measurement of the condition?	Yes	Yes	Yes	Yes	Yes	Yes
Were confounding factors identified	Yes	Yes	Yes	Yes	Yes	Unclear
Were strategies to deal with confounding factors stated?	Unclear	Yes	Yes	Yes	Yes	Unclear
Were the outcome measured in a valid and reliable way?	Yes	Yes	Yes	Yes	Yes	Yes
Was the appropriate statistical analysis used?	Yes	Yes	Yes	Yes	Yes	Yes

In one other study the description of the settings from which the subjects were drawn was unclear [26]. This study used primary data from a previous study and excluded data from communities where greater than 50% of data were missing and it was not explicitly stated, which communities were excluded [26].

### Characteristics of sources of evidence

The final analysis included six studies (Table 3), all quantitative, four from the US, one each from Colombia and Canada, published between 2008–2018. The length of data collection for the studies ranged from six nights [16] to two years [27], and the total number of participants was 27,509. Data for the studies were collected directly from participants at drop-in centres, temporary shelters [25] or solely on the street [16, 28], from existing homelessness databases, [27] and data from previous campaigns including the 100K campaign [26] and Directions Home in Texas [13].

**Table 3:** Data charting vulnerability indices.

Author/year/city country	Title of study	Participants/Data collection	Study Aim	Design	Results: characteristics, medical conditions	Conclusions relating to vulnerability index
Berbesi, Segura, Cardona, Caicedo, (2017) Medellin, Colombia	HIV vulnerability index in homeless persons	N = 338 Data collected first half of 2014, homeless persons on the streets of Medellin (Colombia)	To determine an HIV vulnerability index for homeless persons	Cross sectional study using a HIV vulnerability tool developed by the authors	Age range 18–65 years, 50% of participants were >41 years, 71% (n = 241) single, HIV prevalence was 8.15% (CI 95% 3,92–12,37)	HIV vulnerability is defined as the reduced ability to anticipate (lack of knowledge to protect oneself), resist (risky sexual behaviour and drug use) and recover (lack of social supports), which limits a homeless person’s ability to access HIV prevention and support services
Bowie & Lawson (2018) Seattle, US	Using the Vulnerability Index to Assess the Health Needs of a Homeless Community	N = 46 Data collection over two-months, participants were encountered in drop-in day centres, temporary winter overnight shelter, and on the street in Seattle. All participants met the Federal definition of homelessness	To assess the health status and health service usage of people experiencing homelessness in an urban neighbourhood on the edge of a large city using a vulnerability index	Cross-sectional survey design using interviews to complete the vulnerability index. Interviews conducted by a member of the homeless community paired with a faculty member/ graduate student nurse	Age range 28–66 years, average age 47 years. Majority male (70%), mean continuous duration of homelessness 4.7years, range 1 to 19 years. Common medical conditions: heart disease (37%), skin conditions (28%) and hepatitis C (22%), abuse of drugs or alcohol (78%), mental health condition (44.7%).	Vulnerability index was easy to administer, and effective in compiling a health profile of people experiencing homelessness in the community, enabling workforce planning. Vulnerability index lacks robust psychometric testing so cannot be used for outcome prediction. Vulnerability index lacks a mechanism for interpreting the overall vulnerability score beyond prioritising shelter
Brown, Cummings, Lyons, Carrion, & Watson (2018). Midwest, US	Reliability and Validity of the Vulnerability Index-Service Prioritisation Decision Assistance Tool in real-world implementation	N = 1407 Data retrieved from Homeless Management Information System April 2014-April 2016 VI-SPDAT administered via street outreach	Reliability and validity of the VI-SPDAT	Internal, test-retest and inter-rater reliability and construct and predictive validity of the VI-SPDAT	VI-SPDAT has limitations in its reliability and validity. Test-retest reliability coefficients were below acceptable thresholds. Several items on the Socialisation and Daily Functions and Wellness domains demonstrated negative associations with other variables	Use of the VI-SPDAT in a community context is not recommended as the sole instrument for housing prioritisation, further psychometric testing is required. Total VI-SPDAT score not a predictor in the re-entry to homeless services but correlated with higher risk
Cronley, Petrovich, Spence-Almaguer, & Preble. (2013) Fort Worth, Texas, US	Do Official Hospitalisations Predict Medical Vulnerability among the Homeless? A Postdictive Validity Study of the Vulnerability Index	N = 97 Data from vulnerability index assessment of homeless individuals in 2008. Individuals scoring zero or one on the vulnerability index were not offered housing and not included in the sample. Participants were those who were assessed using the vulnerability index in 2008 and had received health care from the hospital in Tarrant County	Empirical evaluation of the vulnerability index as a tool to assess the degree of medical vulnerability and health service utilisation among people experiencing homelessness	Postdictive validity of the vulnerability index assessment data was paired with the health care utilisation data collected from the Hospital in Tarrant County	Age range 29–69 years mean age 48.69 years, 53.3% African American, 57.7% male. Common medical conditions were heart disease (35.1%), asthma (29.9%), Hepatitis C (23.7%), Tuberculosis (16.5%), Liver disease (16.5%), Emphysema (13.4%). When controlling for gender and race, individuals who scored higher on the vulnerability index accessed hospitals more frequently, compared to those who had a low vulnerability index score	Official hospital records are predictive of overall vulnerability index scores and are correlated with self-reported hospitalisation data but are not predictive of the subcomponents of the vulnerability index, perhaps indicating the underutilisation of health care for those with serious health conditions
Montgomery, Syzmkowiak, Marcus, Howard, Culhane. (2016) 62 communities in the US	Homelessness, Unsheltered Status, and Risk Factors for Mortality: Findings from the 100,000 Homes Campaign	N = 25,489 Total N = 13761 Unsheltered N = 11728 Sheltered Data collected as part of 100,000 Homes Campaign, 2008–2014, in 96 communities	Comparison of characteristics of people experiencing homelessness who were sleeping in unsheltered situations with those who were accessing shelter	Cross sectional survey data collected through the application of the vulnerability index, to assess sheltered status and risk factors for mortality of people experiencing homelessness	Highest proportion of participants were aged between 50–59 years (33.9% vs. 34.3% sheltered vs. unsheltered), majority male (70.2% vs. 75.6% sheltered vs. unsheltered), majority homeless for 1–5 years (47.8% vs. 46.5%, sheltered vs. unsheltered). Common medical conditions, mental health (53.9% vs 53.7%, sheltered vs. unsheltered), living with liver/and or kidney disease (11.8% vs. 15.1%, sheltered vs. unsheltered), HIV/AIDS 3.3% vs. 3.7%, sheltered vs. unsheltered), ever treated for drug/alcohol abuse 45.1% vs 47.2%, sheltered vs. unsheltered)	Unsheltered status correlated with being male, white or mixed race, history of military service, incarceration, foster care, use and treatment for drug and alcohol abuse, less likely to have more than high school education, more likely to receive income from informal sources, higher rates of the high risk conditions measured by the vulnerability index, more likely to live in warmer climates. Sleeping unsheltered had a 12% higher odds of having at least one risk factor for mortality, other correlates for mortality were female, military service, homeless for <5years, prior incarceration. The findings highlight the need to identify those at risk and assist them in their transition from military service or incarceration or foster care, to ensure that they do not become unsheltered and later, at risk of increased mortality
Nicholson, Graham, Emery, Schiff, Giacomin, Tanasescu. (2008) Calgary, Canada	Describing the Health of the Absolutely Homeless Population in Downtown Calgary 2008	N = 132 Data collection October–December 2008, as a street level survey	To describe the health profile of the homeless population in Calgary using the vulnerability index and compare the findings with homeless people in American cities	Survey data collected through the application of the vulnerability index, comparison of findings with homeless people from American cities	Mean age 40 years, 79.5%(n = 105) male, a third identified as Aboriginal (n = 35), average length of homelessness was just under 6 years, 45.5% (n = 60) sleeping most frequently in shelters, 19.6%. 73% reported at least one health condition and 55% had two or more, history of frostbite (25.8%, n = 24), asthma (23.4%, n = 31), hepatitis C (22%, n = 29). 32.6% (n = 43) current or previous treatment for mental health issues, 96.2% (n = 127) reported a substance abuse problem	The vulnerability index tool was effective in assessing the homeless population. Compared with the general population in Canada, participants reported higher incidence of kidney disease, asthma, emphysema and cancer. Females reported higher incidence of cancer, hepatitis C, liver, kidney heart and mental health disease. Homeless people in Calgary have a higher risk of mortality

The aims of the studies varied. One study used a vulnerability index to establish a health profile of a homeless population, [16] one study compared characteristics of ‘unsheltered and sheltered’ people experiencing homelessness using a vulnerability index, [26] another used a vulnerability index to assess health service usage [25]. One study examined the validity and/ or reliability of a vulnerability index [27] and another conducted an empirical evaluation of the predictive capacity of demographic, health and clinical variables on vulnerability score [13] and another developed and examined the validity of an HIV vulnerability index [28].

Where reported, the mean age of participants was 48 years [13, 25] and 40 years [16, 28]. Most participants were male [13, 16, 25] and the majority of participants reported at least one severe medical condition [13, 16, 25]. Mean length of continuous homelessness was reported as 4.7 years [25] and just under six years [16]. The most common medical conditions reported by participants were heart disease and skin conditions, [25] hepatitis C [16, 25] and asthma [16].

### Results of individual sources of evidence

#### Characteristics of vulnerability indices

**Fig 3:**
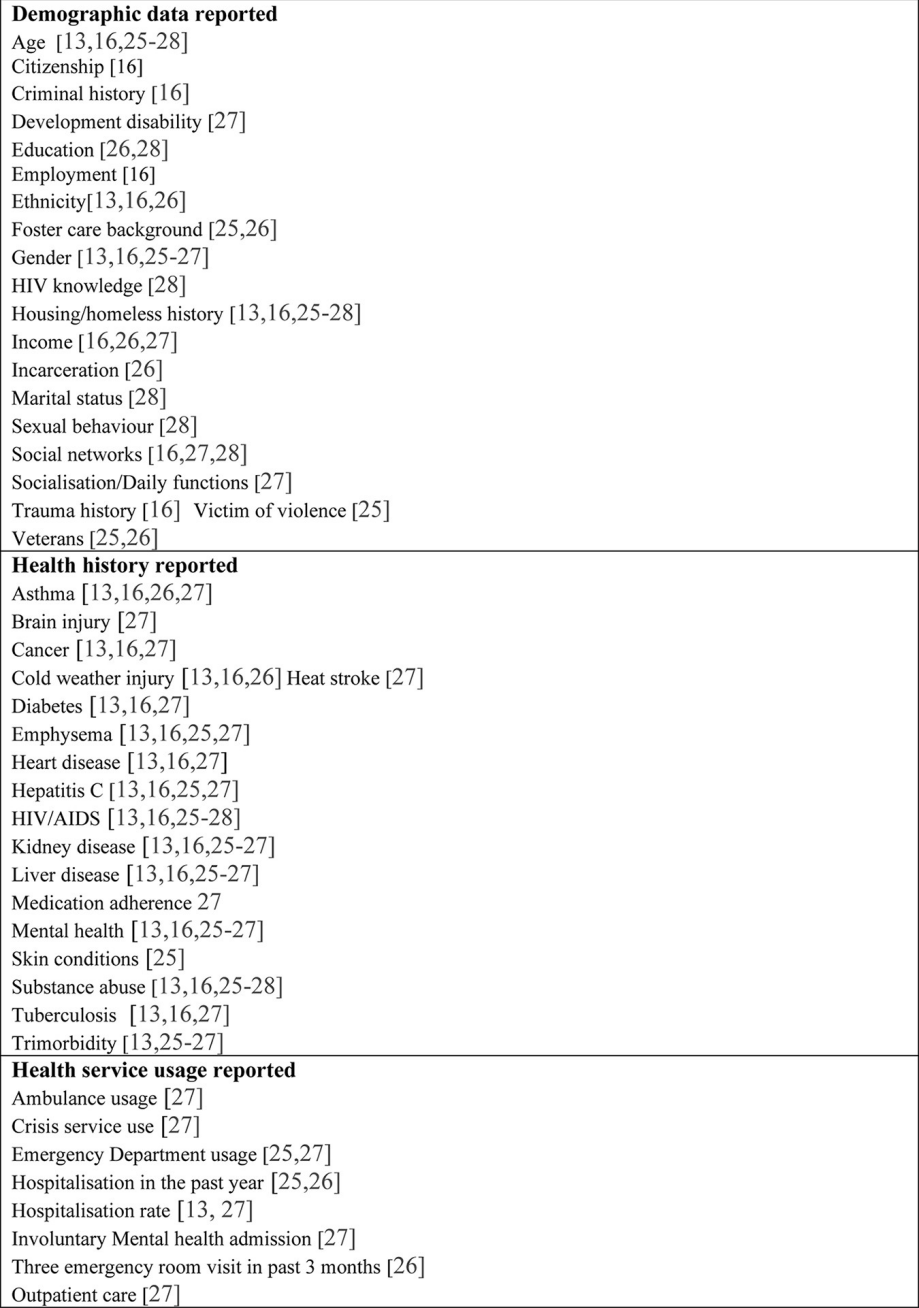
Characteristics of vulnerability indices reported in the included studies.

The included studies used different vulnerability indices and included different characteristics, as shown in Fig 3. Four studies used the original vulnerability index developed through the 100K campaign [12, 13, 16, 25]. One study developed an HIV vulnerability index, [28] to explore the associations between homelessness, social factors and HIV risk behaviours in participants living in Colombia. One study examined the reliability and validity of the Vulnerability Index–Service Prioritization Decision Assistance Tool (VI-SPDAT), a 50-item assessment composed of yes/no questions grouped into four themes: history of housing and homelessness, risks, socialisation and daily functions and wellness. In addition to these domains there is one demographic question to assess age. The responses to the domains are scored and subtotalled to an overall score of 0 to 20 [27].

The health history reported through the four studies using the original vulnerability index was similar and included cold injury (frostbite) [13, 16, 25, 26]. The study of the VI-SPDAT elicited participants’ history of frostbite and/or heat stroke [27] as well as detailed health history including tri-morbidity and an assessment by the surveyor of the participant’s level of hygiene and any signs of serious health conditions. The term ‘tri-morbidity’ is understood as the combination of a serious physical health condition, serious mental health condition and drug and alcohol dependence [29]. The study of the HIV vulnerability index reported sexual history, participant’s knowledge of HIV transmission, drug use frequency and social networks as well as participants’ perceptions of rejection and discrimination [28]. Based on their findings the authors developed a model for HIV vulnerability that includes components of risky behaviours (e.g. drug use, no condom use), lack of support and presence of rejection by others in the form of discrimination or stigmatisation, and erroneous knowledge of HIV and AIDS [28].

The health service usage reported by each of the studies varied, reflecting the different aims of the studies. For example, in their examination of the reliability and validity of the VI-SPDAT, Brown and colleagues (2018) elicited detailed data relating to the frequency of participants’ attendance to ED, crisis services, inpatient services, ambulance attendances, treatment for drug or alcohol dependence, and mental health review in the past six months. Two studies did not report health service use [16, 28].

#### Health outcomes achieved by applying a vulnerability index

While none of the studies identified any specific health outcomes achieved by applying a vulnerability index, the studies highlight specific risk factors for increased morbidity and mortality, such as being female, prior military service, continuous homelessness of greater than five years, prior incarceration, lower levels of education and a history of substance use [26]. The study of the HIV vulnerability index identified risky behaviours and social factors including a lack of social support, rejection and discrimination, limiting the decision-making ability of study participants and thereby increased their risk of exposure to HIV [28].

### Reported limitations of vulnerability indices

The included studies identified limitations of using vulnerability indices. Studies using the original vulnerability index highlighted the self-report nature of data collection as a limitation due to the potential subjectivity of responses [13, 25, 26]. The face to face administration of vulnerability indices was also identified as having the potential to impact on the transparency of responses [25]. In the Bowie & Lawson (2018) study none of the participants reported a diagnosis of HIV, and the authors believed this was unlikely to be valid and more likely a symptom of fear of disclosure. Furthermore, the potential to misunderstand the terminology used in a vulnerability index was also identified. For example, the original vulnerability index uses the term ‘heart disease’ and whilst some participants in the Bowie & Lawson (2018) study had hypertension they did not self-report it because they were not aware that hypertension would be included under the term heart disease.

Another limitation highlighted was the predictive capacity of the original vulnerability index. In their postdictive validity study of the vulnerability index, Cronley and colleagues (2013) suggest that their findings offer ‘…tentative support…’ for the validity of the vulnerability index and its capacity to predict hospital admissions, in so far as individuals who scored higher on the vulnerability index had more frequent hospital admissions [13]. Although, in relation to the self-reporting of chronic physical health conditions, mental health and substance use, hospital records were not a significant predictor of an individual’s vulnerability index score [13]. The authors conclude that further research is required to assess the accuracy of relying on self-reported health status [13].

The authors using the VI-SPDAT reported a tendency for participants to over-report and recommended triangulation of sources of data other than self-report [27]. The authors called for further psychometric testing and did not recommend the VI-SPDAT as the sole instrument for housing prioritisation [27].

### Synthesis of results

In relation to the research questions posed by this scoping review, the characteristics included in the vulnerability indices used in the studies, the most common demographic characteristics were age and housing status/history, most commonly included health history characteristics were trimorbidity, substance use, mental health, liver, kidney and heart disease, emphysema and asthma, cold weather injury, diabetes and cancer. There were no specific health outcomes reported from applying any of the vulnerability index tools, as this was not an aim of any of the included studies.

## Discussion

### Summary of evidence

This paper reports a scoping review of the application of vulnerability indices to people experiencing homelessness. In developing and implementing a tool for the Australian context, we identified three translational findings from this review.

Our first finding is that the breadth of characteristics included in existing vulnerability indices suggest that an index can be tailored to the context and service in which it will be used [28]. The most common health conditions identified in participants in the included studies were heart disease, skin conditions, hepatitis C and asthma. These conditions are also prevalent among people experiencing homelessness in Sydney. In addition, we would consider including traumatic brain injury (TBI) and heat stroke, which are already included in the VI-SPDAT [27]. Often secondary to unintentional injury [9], the lifetime prevalence of TBI is high among those experiencing homelessness and associated with poorer health outcomes and higher suicidality [30].

Secondly, our project requires that we develop a vulnerability index designed specifically to prioritise clients for access to health care. Both the VI-SPDAT and the original vulnerability index have been successfully applied to homelessness services across Australia to prioritise clients for housing [25, 27]. Now in its third iteration, the VI-SPDAT appears to be the most popular assessment tool in Australia, and is used by Micah Projects, Brisbane [31] Australian Alliance to End Homelessness [32], Homelessness NSW [20] and the Western Australia Initiative [33].

Thirdly, the tool needs appropriate psychometric testing. Whilst proven valuable as a data collection tool, the capacity of the original vulnerability index to predict outcomes is less certain [13, 25, 26]. Following their empirical evaluation, Cronley and colleagues [13] suggest the vulnerability index would be more effective if used in conjunction with other tools that collect data on social supports and health behaviours regarding vulnerability, rather than used on its own. Analysis of the reliability and validity of the VI-SPDAT, the original vulnerability index by the US Department of Housing and Urban Development and the National Alliance to End Homeless concluded that the vulnerability index and VI-SPDAT are limited in their ability to select the best intervention and predict the most successful intervention [34].

### Recommendations for future research

The overall findings of this scoping review demonstrate the use of vulnerability indices as tools to identify the health status of homeless populations. In our St Vincent’s Hospital project, we have gained permission to modify the VI-SPDAT to develop a specific health version of this existing tool.

### Limitations

This scoping review has some limitations. Using articles and databases in English language meant relevant non-English studies were not identified. The search was limited to peer-reviewed publications. In the search there were several government reports identified, and these were excluded. The sample of identified studies was relatively small. Only six studies met the inclusion criteria for this review, indicating the need for more peer-reviewed publications that apply a vulnerability index to homeless populations.

## Conclusion

People experiencing homelessness are known to be vulnerable to poor health outcomes. Vulnerability indices are a proven method of gaining data on the health status and health needs of people experiencing homelessness. In developing and implementing a vulnerability index as part of this St Vincent’s Hospital project, the findings of this scoping review indicate that existing indexes have been tailored to the needs of the population to which it is to be applied. Furthermore, conducting thorough psychometric testing is important so that an index can be used as an accurate predictor of risk of mortality and morbidity.

## Supporting information

S1 Checklist PRISMA checklist. (PDF)

S1 File Search strategy. (DOCX)

## Abbreviations

LGA: local government area
ABS: Australian Bureau of Statistics
VI-SPDAT: Vulnerability Index–Service Prioritization Decision Assistance Tool
