# Supplementary material for: Scoping review of the characteristics assessed by vulnerability indices applied to people experiencing homelessness
Source: PLoS One. 2021 Jul 9;16(7):e0254100. doi: 10.1371/journal.pone.0254100 (PMC8270124; doi:10.1371/journal.pone.0254100)
Supplement: S1 File — (DOCX) [file pone.0254100.s002.docx]

| **21/2/20** | |  |  |  |
| --- | --- | --- | --- | --- |
| **Databases** | “Vulnerability Index*”AND  Homeless* | “Vulnerability Index*” AND  Aboriginal* | | “Vulnerability Index*” AND Homeless* AND Aboriginal* OR “Torres Strait Islanders*” OR Indigenous* OR” First Nation People*” OR Tribal* OR ATSI* OR Aborigines* |
| Scopus | 28 | 4 | | 1 |
| Medline | 3 | 0 | | 8 |
| EMBASE | 3 | 0 | | 0 |
| PubMed | 10 | 2 | | 0 |
| Web of Science | 2 | 0 | | 1 |
| CINAHL | 2 | 0 | | 0 |

Search Strategy

Searched 21^st^ February 2020.

Searched 21^st^ February 2020.

| Database | Conroy, E AND Homeless* | O’Connell, J AND Homeless* | Parsell, C AND Homeless* | Teasdale, M AND Homeless* | Baldry , E AND Homeless* |
| --- | --- | --- | --- | --- | --- |
| Scopus | 24 | 32 | 46 | 0 | 0 |
| Medline | 5 | 52 | 7 | 0 | 2 |
| EMBASE | 3 | 3 | 4 | 0 | 2 |
| Cochrane | 0 | 0 | 0 | 0 | 0 |
| PubMed | 3 | 37 | 7 | 0 | 2 |
| Web of Science | 11 | 48 | 31 | 0 | 6 |
